# Supplementary material for: When Is the Right Moment to Pick Blueberries? Variation in Agronomic and Chemical Properties of Blueberry (Vaccinium corymbosum) Cultivars at Different Harvest Times
Source: Metabolites. 2022 Aug 26;12(9):798. doi: 10.3390/metabo12090798 (PMC9502264; doi:10.3390/metabo12090798)
Supplement: Supplementary file 1 [file metabolites-12-00798-s001.zip › metabolites-1833490-supplementary.pdf]

Table S1. Chemical parameters used for Principal component analysis

No.	Chemical parameters	D1	D2	D3	D4	C1	C2	C3	C4	BC1	BC2	BC3	BC4
1	RSA	27.64	29.66	27.78	24.83	25.91	24.71	26.02	26.16	29.07	29.05	29.98	29.02
2	TPC	2.52	2.52	2.35	1.45	1.78	1.64	1.74	1.61	2.51	2.21	1.86	1.98
3	TAC	1.65	1.75	1.61	0.49	0.97	0.78	0.96	0.73	1.34	0.95	0.56	0.71
4	Aesculin	0.467	0.661	1.276	0.302	0.455	0.277	0.297	0.348	0.463	0.774	0.398	0.440
5	p-Hydroxybenzoic acid	0.000	0.000	0.000	0.000	11.048	0.970	4.693	6.058	3.407	4.080	8.238	6.702
6	5-O-Caffeoylquinic acid	6.405	2.789	2.191	8.737	3.746	34.793	3.241	7.584	3.202	6.493	9.982	3.589
7	Caffeic acid	0.344	0.283	0.566	0.285	0.709	0.318	0.284	0.187	0.552	0.279	0.271	0.449
8	Aesculetin	0.378	0.451	0.630	0.555	0.833	0.230	0.252	0.356	0.549	0.330	0.419	0.569
9	Rutin	6.956	6.043	4.780	25.642	16.367	24.612	24.277	22.736	5.644	21.309	26.254	21.425
10	Quercetin 3-O-glucoside	17.239	17.432	13.985	21.462	18.261	22.717	24.354	22.449	12.012	22.406	23.189	16.110
11	Ellagic acid	0.000	0.000	0.000	0.000	0.708	0.000	0.298	0.000	0.000	0.000	0.000	0.000
12	Kaempferol 3-O-glucoside	0.410	0.471	0.327	5.127	3.392	5.767	7.151	6.575	0.256	5.229	6.041	4.816
13	Quercetin 3-O-rhamnoside	0.000	0.000	0.000	0.000	7.318	15.452	12.307	13.912	0.000	12.174	12.395	5.929
14	Phlorizin	0.309	0.236	0.179	0.091	0.143	0.159	0.167	0.137	0.338	0.288	0.140	0.207
15	Quercetin	62.995	85.858	119.136	69.045	130.781	47.120	90.864	57.267	136.391	81.966	72.343	92.895
16	Kaempferol	1.032	1.306	1.191	2.409	2.109	2.042	2.397	1.913	1.109	1.996	2.081	2.054
17	Isorhamnetin	2.857	5.023	6.999	0.752	3.927	1.127	0.956	0.863	6.905	1.471	0.700	1.257
18	Sorbitol	3.781	1.872	0.608	0.842	0.730	0.712	0.485	1.044	0.412	0.734	1.256	0.825
19	Trehalose	0.019	0.001	0.004	0.009	0.003	0.002	0.002	0.004	0.001	0.003	0.000	0.001
20	Glucose	44.471	39.859	24.708	27.749	26.864	26.516	22.424	24.586	28.632	24.809	38.287	36.775
21	Fructose	32.659	28.124	15.159	19.197	33.680	33.978	27.080	24.908	29.396	24.993	37.820	31.960
22	Sucrose	2.267	1.564	1.209	1.097	1.844	1.731	0.922	1.015	0.682	0.850	1.023	1.071
23	Isomaltose	0.077	0.003	0.002	0.001	0.002	0.001	0.001	0.001	0.002	0.003	0.002	0.001
24	Turanose	0.088	0.125	0.038	0.002	0.002	0.002	0.003	0.003	0.005	0.008	0.003	0.004
25	Gentiobiose	0.002	0.002	0.001	0.000	0.000	0.000	0.000	0.001	0.000	0.000	0.000	0.000
26	Maltose	0.144	0.061	0.026	0.028	0.071	0.027	0.017	0.049	0.123	0.196	0.073	0.126
27	Maltotriose	0.036	0.025	0.018	0.031	0.073	0.038	0.037	0.090	0.028	0.057	0.020	0.329
